# Does work passion benefit or hinder employee’s career commitment? The mediating role of work–family interface and the moderating role of autonomy support

**DOI:** 10.1371/journal.pone.0269298

**Published:** 2022-06-03

**Authors:** Yeseul Jung, Young Woo Sohn

**Affiliations:** 1 Psychological Science Innovation Institute, Yonsei University, Seoul, South Korea; 2 Department of Psychology, Yonsei University, Seoul, South Korea; Universiti Pertahanan Nasional Malaysia, MALAYSIA

## Abstract

Based on the dualistic model of passion, this study explored the relationship between distinct types of work passion and career commitment, as well as the mediating role of work–family interface and the moderating role of autonomy support. A two-wave study was conducted among South Korean workers (N = 250) over a 5-month time period. Results showed that harmonious work passion was positively associated with career commitment, whereas obsessive work passion was not significantly associated with career commitment. Moreover, work–family enrichment partially mediated the positive effect of harmonious work passion on career commitment, and work–family conflict fully mediated the negative effect of obsessive work passion on career commitment. Results further indicated that autonomy support strengthens the positive effect of harmonious work passion on work–family enrichment. Furthermore, this study expanded the understanding of the underlying psychological mechanisms of effects of work passion. The implications and limitations of the study and potential topics for future research are discussed.

## Introduction

Scholars have increasingly focused on work passion as an important factor impacting work-related attitudes and behaviors in the organizational psychology literature [1–3]. Work passion is defined as a strong inclination or desire toward work-related activities that people like, whereby they experience the meaning of work, and wherein they spend a great deal of time and effort to achieve their work-related goals [3,4]. Since work is a central part of employees’ life domain and a pivotal feature of their identity [4,5], being passionate about work can be a key factor influencing not only their work and career outcomes but also their life well-being.

According to the dualistic model of passion [3], passion can be classified into two subdimensions: harmonious and obsessive passion. Harmonious passion refers to an autonomous internalization of an activity into one’s identity, while obsessive passion refers to a controlled internalization of an activity into the person’s identity due to an irrepressible pressure [3,4]. Previous research indicates that these differences between passion types result in various outcomes. Harmonious passion is associated with adaptive consequences, such as psychological well-being [6,7], job satisfaction [8], engagement [9], and performance [10]. Obsessive passion is associated with maladaptive consequences, such as psychological distress [11], burnout [8,12], cynicism [13], and intrapersonal conflict [14].

Despite numerous studies on work passion, few studies have examined the impact of work passion as a dispositional and motivational factor in work- and career-related domains. Previous research on work passion has various limitations. Although work passion is closely related to career-related outcomes, such as career satisfaction, career commitment, and career decision-making self-efficacy [13,15], limited research has focused on work passion as a predictor of career-related outcomes. Work passion serves as a motivational resource in promoting individuals’ professional growth and development [15]. Drawing on the dualistic model of passion [3], employees who are highly passionate toward their work strongly identify with and feel motivated to engage in it; therefore, they may experience greater career commitment. Based on a meta-analytic review of career commitment, work passion as a motivation attribute can be a predictor of career commitment [16]. In particular, following the dualistic model of passion, work passion can be pivotal in fostering or hampering career commitment. Research has revealed that employees with harmonious work passion experience higher levels of career commitment than those with obsessive work passion [1,17]. Therefore, we may expect that the two types of work passion relate differently to career commitment.

Furthermore, few studies have explored why and how work passion relates to career commitment. Several researchers have suggested work–family interface to be an intervening variable between work-related resource and demand and employees’ outcomes [18–21]. In line with the job demands–resources (JD-R) theory [22], work passion—as a work-related personal resource and demand—can have opposing influences on an important employees’ outcome–career commitment through the work–family interface as a motivational and an impairment process [21]. Accordingly, harmonious work passion as a personal resource can enhance career commitment through a motivational mechanism, work–family enrichment, while obsessive work passion as a personal demand can reduce career commitment, through an impairing mechanism, work–family conflict. Moreover, preliminary evidence suggests that work passion affects work and career-related outcomes via the work–family interface because an individual’s work and family life are closely intertwined [23,24]. Despite the fact that work–family interface is a psychological mechanism explaining the relationship between work passion and outcomes [21,25], the mediating role of the work–family interface—linking work passion to career commitment—remains under researched. Thus, integrating the JD-R theory and dualistic model of passion, we consider work–family enrichment and work–family conflict as mediators to investigate the dual-pathway—the motivation and impairment paths that intervene in the two distinct types of work passion to career commitment.

Finally, although the effects of work passion can vary depending on the specific context [10,26], research has yet to examine the situational or contextual factors strengthening the positive effect and weakening the negative effect of work passion. Given that creating a supportive work environment may reinforce the positive effect of passion in work contexts, understanding the boundary conditions where work passion can increase or diminish work-related outcomes is necessary. Autonomy support is an appropriate factor for assessing situational strength that affects the positive effects of passion on an employee’s work outcomes [27,28]; accordingly, we explore autonomy support as a potential moderator in the relationship between work passion and work–family interface. For example, research has found that in autonomy-supportive work environments, employees with harmonious passion are more likely to have ideas for work improvement and intention to give voice to their thoughts [27]. Further, organizational environments that provide substantial freedom, such as an empowering environment, nourish the influence of a harmonious work passion on an employee’s innovative work behaviors [28]. Therefore, we assume that a contextual workplace resource (i.e., autonomy support) can act as a moderating variable benefiting or hampering the effects of work passion on a better-quality work experience.

To address the aforementioned research gaps, we investigate the underlying psychological mechanisms of work passion on career commitment. Specifically, by applying the dualistic model of passion [3], we clarify the differential impacts of the two types of work passion on career commitment. Furthermore, we examine the mediating roles of the work–family interface—that is, work–family enrichment and work–family conflict—on the link between work passion and career commitment. Finally, we explore the moderating role of autonomy support—an organizational contextual factor—on the relation between work passion and work–family enrichment and conflict, which enhances its benefits and mitigates its adverse outcome. Thus, this study contributes to a deeper understanding of how and why work passion relates to career commitment, thereby extending the theoretical framework of a dualistic model of passion in the work setting.

## Theory and hypotheses

### The dualistic model of passion

Work passion refers to an individual’s strong inclination to invest substantial time and energy in their work-related activities that they like and consider important [3]. The dualistic model of passion [3,4] distinguishes work passion into harmonious and obsessive passion in terms of how the passionate activity has been internalized into individual’s identity. Harmonious work passion arises from individuals’ identity formed by an autonomous internalization of work-related activities, and it freely accepts the activities important for them without attachment to any external contingencies [3]. Employees with harmonious work passion are not forced to engage in the work-related activity; rather, they autonomously choose to do so [4,29]. This activity does not overpower space in individuals’ identity and is in harmony with other facets of their lives, such as family, leisure, and social lives [4,29]. For example, workers with harmonious passion enjoy and value their job, but they may turn off work mode after work and focus on non-work activities, such as relaxing, socializing with friends, or fulfilling family responsibilities [21,30]. Conversely, obsessive work passion derives from the individuals’ identity formed by a controlled internalization of work-related activities [4]. Obsessively passionate individuals feel compelled to engage in the activity under external control, such as intra and/or interpersonal pressure [4]. This may lead the activity to occupy disproportionate space in the individuals’ identity, thus conflicting with other activities in their lives [3]. For instance, workers with obsessive passion feel compelled to engage in work even after work hours, which may hinder them from enjoying their leisure time and contributing to their family duties [21].

Based on the dualistic model of passion, previous studies have reported that harmonious passion is closely related to positive work outcomes (e.g., job satisfaction and organizational commitment [1,2]); conversely, obsessive passion is not related to positive work outcomes (e.g., work satisfaction [30] and work performance [2]) but to negative ones (e.g., exhaustion and cynicism [13] and burnout [8]). Specifically, because employees with harmonious work passion present flexible forms of involvement in their work-related activity, they are more likely to experience positive affect and high levels of task engagement [31], flow [3], vitality [11], and objective performance at work [10]. However, employees with obsessive work passion display rigid persistence toward work-related activities, which further promotes internal compulsion to engage in work regardless of the personal costs and risks, leading to failed work commitments and task completion as well as damaged interpersonal relationships in the organization [3,30]. Hence, they are more likely to experience negative affect and difficulty concentrating on the task [3]. Furthermore, they tend to have lower levels of vitality [9] and objective performance at work [10] and higher levels of cynicism, psychological distress, and emotional exhaustion [26].

### The relationship between work passion and career commitment

Career commitment is defined as an individual’s overall attitude toward their career or occupation [32]. It describes the extent of an individual’s motivation to fulfill the preferred career role [33], including their persistence in pursuing career goals despite any obstacles and adversities encountered [34]. Considering that career commitment is characterized by the extent to which individuals identify with their career [32,35], work passion is expected to be associated with career commitment.

Following the logic of the dualistic model of passion, work passion contributes to individuals’ commitment to their career or occupation through the internalization of the object of passion, such as career [1,15]. As individuals with high autonomous self-structure, such as harmonious passionate workers, pursue learning and growth goals [25,36], they may engage in proactive and adaptive career behaviors and achieve their career goals [37]. Furthermore, individuals with harmonious work passion have higher levels of psychological career resources, reflecting individuals’ career consciousness and mindfulness of important self-regulatory resources that enhance proactive career self-management [38,39]. This leads to greater career development such as career satisfaction [39,40], work meaningfulness [40], work engagement [41], and career resiliency [42]. By contrast, individuals with obsessive work passion tend to perceive a lack of career resources or emotional energy [43] because they experience internal pressure while pursuing career goals. This may cause them to experience difficulty in committing to their career goal.

Prior research has indicated that the two types of work passion have differential effects on career commitment. Harmonious passion produces stronger relationships with career commitment, promotability, career decision-making self-efficacy, and career-persistence intention, than does obsessive passion [15]. Moreover, harmonious passion is associated with a higher sense of control over one’s career and pursuit of the same career choice if given the opportunity, than is obsessive passion [11]. In addition, Burke et al. [1] suggested that employees with harmonious work passion exhibit relatively higher career satisfaction and career commitment than those with obsessive work passion in the Chinese samples.

**Hypothesis 1a**: Harmonious work passion is positively related to career commitment.
**Hypothesis 1b**: Obsessive work passion is negatively related to career commitment.

### The relationship between work passion and work–family interface

Work–family enrichment is defined as the degree to which experiences gained through work role improve engagement and the quality of life in the family role [44]. Work–family conflict refers to “a form of inter-role conflict, in which role pressures from work and family domains are mutually incompatible in some respect” [45].

We believe that the JD-R theory [46] provides an appropriate theoretical framework for explaining the effects of work passion on work–family interface. According to the JD-R theory, job resource represents aspects of the work that are associated with accomplishing work goals, dealing with work demands, and promoting personal growth and development, while job demand refers to aspects of the work that are associated with physiological and/or psychological costs, including work overload, work pressure, and role ambiguity [22,46]. From this perspective, harmonious work passion can be considered as a work-related resource [21] as it helps foster self-determined motivation [47], maintain control over the work-related activity [3], and achieve work goals [12]. Hence, harmonious work passion creates harmony with multiple life roles and enhances work–family enrichment, leading to the motivational path of such work–family enrichment [21,48]. Conversely, obsessive work passion can be considered as a work-related demand [21] because it increases the internal urge to engage in work [49], overinvest in the work role [23], and continue job-related thoughts after work [25]. Thus, obsessive work passion results in resource depletion and conflict with other life activities and increases work–family conflict, leading to the impairment path of work-family conflict [21,48].

Furthermore, based on the dualistic model of passion, the two types of work passion can differently predict work–family interface. Because of autonomous motivation, individuals with harmonious work passion do not feel compelled to perform the preferred activity; instead, they highly commit to this activity, and they also maintain harmony with other life domains [3]. Thus, those with harmonious work passion experience more positive affect and satisfaction with work-related activity [3,11]. Additionally, they may have positive affect and attitudes in other domains through positive spillover effects. As individuals with harmonious work passion have a high level of self-determined motivation, they are likely to experience high autonomy under certain circumstances that require them to perform multiple roles simultaneously [3,23]. This may facilitate improved handling of work and family role demands. Moreover, employees having harmonious work passion perceive greater environmental and job resources that facilitate activity engagement and commitment [50]. Therefore, they tend to present a high level of work–family enrichment in a multiple role situation [51].

Conversely, with controlled motivation, individuals with obsessive work passion feel excessive internal pressures, struggle with activity engagement, and experience conflict with other life domains [3]. Thus, those with obsessive work passion experience more negative affect and dissatisfaction with work-related activity [3,4]. They may have negative affect and attitudes in other domains through negative spillover effects. Additionally, as obsessively passionate employees perceive greater job demands and obstacles—that may threaten the quality of their work–life experience [50]—they tend to exhibit a high level of work–family conflict in a multiple role situation [51].

Prior research has demonstrated that the two types of work passion differentially predict the experience of the work–family interface. Caudroit et al. [52] found that teachers with harmonious passion arrange their work schedule flexibly and decline extra work after regular work hours, thus experiencing lower work–family conflict. Conversely, teachers with obsessive passion have difficulty in resisting extra work during the weekend or holidays, resulting in more conflict between their work and family roles. Chummar et al. [23] investigated the relationship between the two types of work passion and work–family interface. They proposed that harmonious passion is positively associated with work–family enrichment, while obsessive passion is positively associated with work–family conflict. Accordingly, we hypothesize the following:

**Hypothesis 2a**: Harmonious work passion is positively related to work–family enrichment.
**Hypothesis 2b**: Obsessive work passion is positively related to work–family conflict.

### The relationship between work–family interface and career commitment

Role accumulation theory [53,54] and conservation of resources (COR) theory [55] address why work–family enrichment and work–family conflict contribute to or hinder career commitment. Role accumulation theory suggests that when individuals participate in multiple roles, various rewards (e.g., role privileges, status enhancement, and resources) can be expanded. Hence, performing a role in one domain may generate energy and resources in another domain. Furthermore, COR theory proposes that individuals are motivated to acquire and maintain resources, including personal characteristics, objects, conditions, or energies. In a multiple role situation, performing each role can provide resources that may help them reduce the likelihood of strain and cope with other demands [55]. Both role accumulation and COR theories can be applied to the process of work–family enrichment in that performing multiple roles make individuals invest their resources in another domain, such as career, by perceiving the higher resources and energies. This may subsequently facilitate commitment to their career goals.

Prior studies have demonstrated that positive synergy from work to family, such as work–family enrichment, contributes to higher career commitment [16,56]. Gordon et al. [57] argued that positive spillover from work to family enriches personal resources, self-esteem, and role performance, which in turn results in increased commitment to the organization and satisfaction with job and career. Considering this mechanism, the higher the level of work–family enrichment, the more employees commit to their career with adequate resources and energies acquired in both work and family domains. Therefore, they pursue their career goals to achieve career advancement and success.

Applying both theories to the process of work–family conflict suggests that individuals are motivated to avoid the loss of resources and preserve them. In a multiple role situation, perceiving the higher role incompatibility may increase role strain and conflict [58,59]. Thus, performing multiple roles increases individuals’ perception of the loss of resources, and it reduces time and effort for the achievement of their career goals and development [60]. Consequently, this may lead to a decrease in commitment to their career goals.

Several researchers have found that the work–family conflict is negatively related to career satisfaction and career commitment [61,62]. Negative spillover from work to family creates resource depletion, role overload, and negative affectivity regarding family role-related self-efficacy, which in turn results in lower work engagement and maladaptive career outcomes (e.g., career development, promotability, salary, and turnover) [16,63]. Okurame [62] also indicated that the stress and strain that occurred by work–family conflict negatively affect individuals’ perception of their career in a dual role situation [64]. Therefore, individuals invest less time and effort in their career development, which can result in a lower level of career commitment [62]. Accordingly, we hypothesize the following:

**Hypothesis 3a**: Work–family enrichment is positively related to career commitment.
**Hypothesis 3b**: Work–family conflict is negatively related to career commitment.

### The mediating role of work–family interface

By integrating the JD-R theory and the dualistic model of passion, we expect that work–family enrichment and work–family conflict, as motivational and impairment paths, respectively, will act as mediators for the impact of work passion on career commitment. In particular, individuals with high harmonious work passion are likely to experience more self-determined work motivation, positive affect, and satisfaction with their job, and they can experience harmony in other life domains [4]. These characteristics of harmonious work passion may increase work–family enrichment, which benefits from the positive synergy by performing work and family roles under the dual role situation. This may lead to individuals’ increased resources and energies to invest in the achievement of career goals. Therefore, it might positively influence career commitment.

In contrast, individuals with obsessive work passion are likely to feel pressured to work hard [4]. With the loss of personal control on work, they may exhibit more negative affect and maladaptive attitudes [4]. Such characteristics of obsessive work passion may increase the work–family conflict and imbalance between the work and family domains under the dual role situation. The perception of work–family conflict may lead to the depletion of resources and energies. Eventually, this may negatively influence career commitment. Based on the foregoing paragraph, we propose the following hypothesis:

**Hypothesis 4a**: Work–family enrichment mediates the relationship between harmonious work passion and career commitment.
**Hypothesis 4b**: Work–family conflict mediates the relationship between obsessive work passion and career commitment.

### The moderating role of autonomy support

Autonomy support refers to a contextual factor that facilitates the fulfillment of the individual’s need for autonomy [65]. Autonomy support offers employees authority and choice, encourages self-initiation, and acknowledges their feelings and perspectives [66].

According to self-determination theory, employees with high autonomy need satisfaction are likely to feel joy and satisfaction with their work and deal more adaptively with high job demands [67]. They may experience less psychological distress because they view job demands, including role conflict and role overload, as challenging rather than threatening [68]. However, employees with low autonomy need satisfaction are more likely to perceive job demands as threatening and difficult to deal with, and therefore, inefficiently and passively cope with demanding situations [68]. These individuals are vulnerable to psychological distress when job demands, such as role ambiguity, role conflict, and role overload, are high [67].

Prior research has suggested that work contextual factors, such as supervisor support, a supportive organizational culture, and workplace flexibility, can potentially boost the positive effect of harmonious work passion or buffer the negative effect of obsessive work passion [21,48]. So far, no empirical study has investigated the moderating role of autonomy support on the link between work passion and the work–family interface. However, several researchers have studied the effects of job autonomy on work–family enrichment and work–family conflict [69–71]. For example, Grzywacz and Butler [69] identified that an increase in autonomy promotes the experience of positive spillover from work to family, in turn resulting in greater work–family facilitation and less work–family conflict. Furthermore, a supportive organizational environment is likely to enhance work–family enrichment and undermine work–family conflict [71–74]. According to Hong et al. [74], perceived organizational support contributes to increased internal motivation, which subsequently reduces work–family conflict. Au and Ahmed [72] demonstrated that supervisory support within the organization maximizes resource gain through work–family enrichment and minimizes resource losses through work–family conflict.

Based on the prior studies, autonomy support can act as a resource acquired in the workplace, which enhances work–family enrichment and diminishes work–family conflict. Thus, we can expect autonomy support to strengthen the positive impact of harmonious passion on work–family enrichment and attenuate the negative impact of obsessive passion on work–family conflict. We therefore hypothesize the following:

**Hypothesis 5a**: Autonomy support moderates the relationship between harmonious passion and work–family enrichment.
**Hypothesis 5b**: Autonomy support moderates the relationship between obsessive passion and work–family conflict.

**Fig 1:**
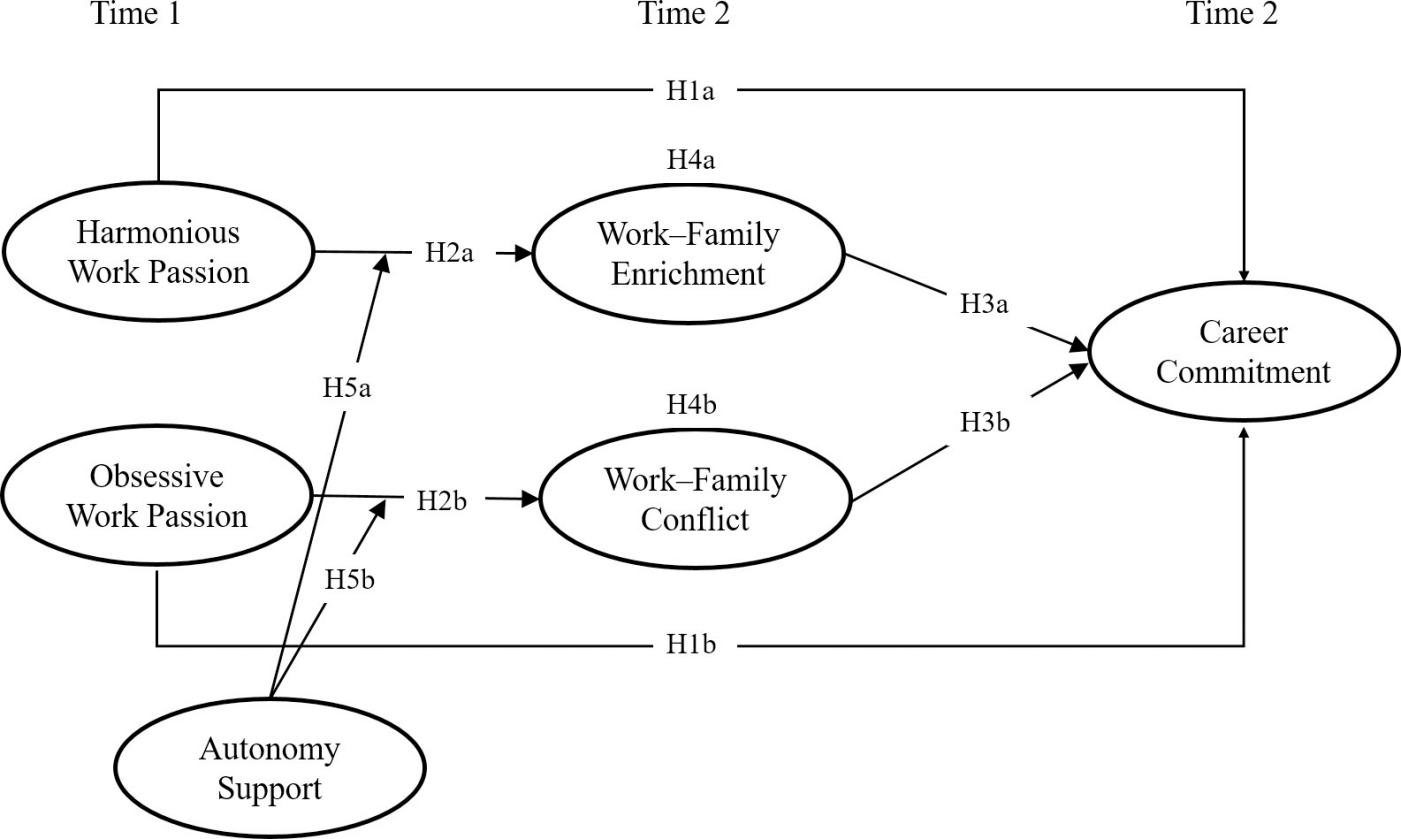
The hypothesized research model.

Based on the above discussion, Fig 1 presents the hypothesized research model:

## Materials and methods

### Procedures and participants

Korean employees, who were over 18 years of age and had more than 6 months of work experience, participated in the current study. Participants were recruited via Invight, an online survey platform, using invitation-based panel. A total of 500 employees completed the questionnaire at Time 1, and 250 of these completed the follow-up questionnaire at Time 2, representing a 50% response rate. Personalization in the form of an e-mail invitation increased the survey’s response rate. Two follow-up reminders were also sent two days after the initial Time 2 invitation. The personalization of invitations and the number of contacts proved effective in improving the response rate [75,76]. As a result, 250 employees were included in our final analyses. At Time 1, we assessed work passion, autonomy support, and demographics. After five months (Time 2), we measured work–family conflict, work–family enrichment, and career commitment. The 5-month time period was chosen for two reasons. First, despite a lack of consensus regarding the ideal time lag to investigate the longitudinal effects of work passion, organizational psychology research generally acknowledges this time period as being sufficient to detect the change in attitudes from Time 1 to Time 2 [49,77,78]. Second, this time period allows for not only minimizing participants’ dropouts but also reducing the potential risks of common method biases [79]. The study protocol followed the Declaration of Helsinki. In the processes of institutional and national guidelines, an ethics approval was not required for this study as it neither involves any manipulations or vulnerable subjects nor collects personal identification details. Online informed consent was obtained from the participants who pressed the “I agree” button prior to taking the survey. The participants were informed that participation in the study was voluntary and that they could stop answering at any time. Anonymity and confidentiality were also strictly assured.

Of the participants, 122 (48.8%) were men and 128 (51.2%) were women. The average age was 42.11 (SD = 10.77), and the average organizational tenure was 15.56 years (SD = 10.43). Positions in the organizations were 34.4% entry-, 32.4% intermediate-, 18.0% middle management-, and 11.2% higher management-level and 4.0% others.

### Measures

#### Work passion

We used the 12-item passion toward work scale, adopted from the works Vallerand and Houlfort’s and Lajom et al.’s study [4,15], to measure work passion. The scale comprises two dimensions, harmonious work passion and obsessive work passion, with six items under each dimension. The sample items were “My work is in harmony with the other activities in my life.” (harmonious work passion) and “I have almost an obsessive feeling for my work.” (obsessive work passion). All items were rated on a 7-point Likert scale ranging from 1 (strongly disagree) to 7 (strongly agree). Cronbach’s alpha for harmonious work passion was 0.93 and that for obsessive work passion was 0.86.

#### Work–family enrichment

We used the positive work to family spillover scale developed by Grzywacz and Marks [80] to measure work–family enrichment. A sample item was “The things you do at work help you deal with personal and practical issues at home.” Four items were rated on a 7-point Likert scale ranging from 1 (strongly disagree) to 7 (strongly agree). Cronbach’s alpha for this scale was 0.79.

#### Work–family conflict

We used the work–family conflict scale, taken from Netemeyer et al.’s study [81], to measure work–family conflict. A sample item was “The demands of my work interfere with my home and family life.” Five items were rated on a 7-point Likert scale ranging from 1 (strongly disagree) to 7 (strongly agree). Cronbach’s alpha for this scale was 0.94.

#### Career commitment

We used the seven items from Blau’s [82] career commitment scale. A sample item was “I definitely want a career for myself in this profession.” The items were rated on a 5-point Likert scale ranging from 1 (strongly disagree) to 5 (strongly agree). Cronbach’s alpha for this scale was 0.83.

#### Autonomy support

We measured participant’s perceived autonomy support from their manager using Baard et al.’s [83] work climate questionnaire–short version. A sample item was “I feel that my manager provides me choices and options.” Six items were rated on a 7-point Likert scale ranging from 1 (strongly disagree) to 7 (strongly agree). Cronbach’s alpha for this scale was 0.95.

#### Control variables

Gender and age have been considered predictors of work–family enrichment and conflict in prior studies [70,84,85]. Therefore, we controlled for gender and age in our analyses.

### Data analyses

Statistical analyses were conducted using SPSS 21 and AMOS 21 software. First, we examined reliability, descriptive statistics, and correlations. Second, prior to testing the hypothesized structural model, we conducted a confirmatory factor analysis (CFA) to test the validity of the measurement model. To assess the model fit of the CFA model, we considered multiple fit indices, including the chi-square goodness of fit statistic, comparative fit index (CFI), Tucker–Lewis index (TLI), root mean square error of approximation (RMSEA), and standardized root mean square residual (SRMR). We followed the model evaluation criteria suggested by Byrne [86,87], that is, CFI ≥ .90, TLI ≥ .90, RMSEA < .08, and SRMR < .10. We also considered the standardized factor loadings, construct reliability, and average variance extracted (AVE) to evaluate the validity of the measurement model. Next, we conducted a structural equation modeling analysis to test the hypotheses that work passion is related to career commitment and mediated by the work–family interface. The indirect effects of work–family enrichment and work–family conflict were tested using a bootstrapping method with 5,000 resamples. If the 95% confidence interval (CI) of the indirect effect did not include 0, the significance of the indirect effects was accepted. Lastly, we conducted hierarchical regression analyses to test the moderating role of autonomy support on the link between work passion and the work–family interface. To further illustrate the statistical significance of the interaction effect, we performed a simple slope test as suggested by Aiken and West [88].

## Results

### Descriptive statistics and correlations

Harmonious passion at T1 was positively correlated with work–family enrichment at T2 (*r* = 0.50, *p* < 0.001) and career commitment at T2 (*r* = 0.48, *p* < 0.001). Obsessive passion at T1 was positively correlated with work–family conflict at T2 (*r* = 0.29, *p* < 0.001) and career commitment at T2 (*r* = 0.26, *p* < 0.001). Work–family enrichment at T2 was positively related to career commitment at T2 (*r* = 0.40, *p* < 0.001), and work–family conflict at T2 was negatively related to career commitment at T2 (*r* = −0.24, *p* < 0.001). Autonomy support at T1 was positively related to work–family enrichment at T2 (*r* = 0.36, *p* < 0.001). S1 Table in the Supporting information provides the means, standard deviations, and correlations between the study variables.

### Measurement and structural model

Prior to testing the hypotheses, we conducted a CFA to examine the measurement model. The fit indices for the CFA indicated that the 5-factor model showed a satisfactory fit to the data, χ^2^ = 265.07 (*df* = 125, *p* < 0.001), CFI = 0.95, TLI = 0.94, RMSEA = 0.07, and SRMR = 0.06. This result indicates that the measurement model fits the data well. Further, we assessed the convergent and discriminant validity of the measurement model. Convergent validity was evaluated by the factor loadings of the measurement items (*r* > 0.50) and the values of composite reliability (CR > 0.70). All standardized factor loadings were statistically significant and exceeded 0.50, ranging from 0.58 to 0.92. All construct reliability was above 0.70, ranging from 0.70 to 0.92. Thus, the convergent validity of the latent constructs was achieved. The discriminant validity was evaluated by comparing the values of square roots of AVE to the inter-construct correlations [89]. The square root values of each construct’s AVE were higher than the inter-construct correlations, thereby indicating satisfactory discriminant validity.

To test our hypotheses, we examined the hypothesized models using structural equation modeling. As shown in Table 1, fit indices for the hypothesized partially mediated model were acceptable: χ^2^ = 346.82 (*df* = 156, *p* < 0.001), CFI = 0.94, TLI = 0.93, RMSEA = 0.07, and SRMR = 0.09. The alternative model (a fully mediated model) showed an acceptable fit: χ^2^ = 370.37 (*df* = 158, *p* < 0.001), CFI = 0.93, TLI = 0.92, RMSEA = 0.07, and SRMR = 0.10. The results of model comparison using chi-square difference test [90] indicated that the hypothesized partially mediated model showed a better fit to the data than the alternative model (Δ *χ*^2^ (2) = 23.55, *p* < 0.001). We therefore chose the hypothesized partially mediated model as our final model.

**Table 1:** Results of fit statistics for measurement, hypothesized, and alternative models.

		*χ* ^*2*^	*df*	CFI	TLI	RMSEA	SRMR
1.	Measurement model	265.07***	125	0.95	0.94	0.07	0.06
2.	Hypothesized model	346.82***	156	0.94	0.93	0.07	0.09
3.	Alternative model	370.37***	158	0.93	0.92	0.07	0.10

**Fig 2:**
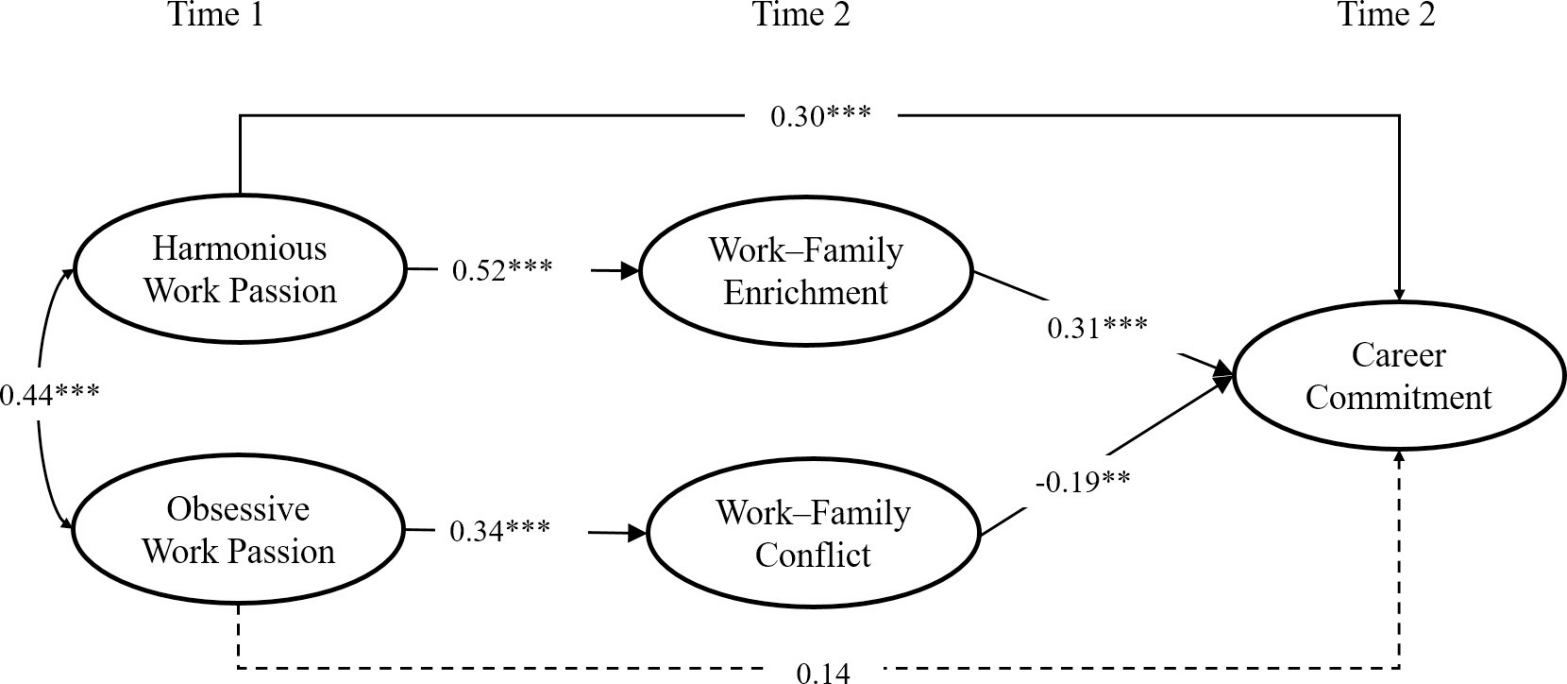
Standardized path coefficients for the hypothesized model. *Note*. Solid lines indicate significant paths and dotted lines indicate non-significant coefficient. The effects of control variables are not shown. **p < 0.01, ***p < 0.001.

Fig 2 presents the standardized path coefficients—associated with Hypothesis 1–3. As Fig 2 indicates, harmonious work passion at T1 was positively related to career commitment at T2 (*β* = 0.30, *p* < 0.001), supporting Hypothesis 1a. Obsessive work passion at T1 was not significantly related to career commitment at T2 (*β* = 0.14, *p* > 0.05), thus not supporting Hypothesis 1b. Consistent with Hypotheses 2a and 2b, controlling for gender and age, harmonious work passion at T1 was positively related to work–family enrichment at T2 (*β* = 0.52, *p* < 0.001) and obsessive work passion at T1 was positively related to work–family conflict at T2 (*β* = 0.34, *p* < 0.001). Supporting Hypotheses 3a and 3b, work–family enrichment at T2 was positively related to career commitment at T2 (*β* = 0.31, *p* < 0.001) and work–family conflict at T2 was negatively related to career commitment at T2 (*β* = −0.19, *p* < 0.01).

Then, we conducted bootstrapping procedures [91] with 5,000 resamples to test the significance of indirect effects of work–family enrichment at T2 and work–family conflict at T2 on the links between harmonious and obsessive passion at T1 and career commitment at T2. The results indicated that the indirect path from harmonious passion to career commitment was significant (bootstrap estimate = 0.12, SE = 0.04, 95% CI [0.06, 0.20]), supporting Hypothesis 4a. The indirect path from obsessive passion to career commitment was significant (bootstrap estimate = −0.05, SE = 0.03, 95% CI [−0.11, −0.01]), supporting Hypothesis 4b. The results supported the hypothesized mediation model.

### Moderation analyses

A hierarchical regression analysis test was performed to analyze the moderating effects of autonomy support on the relationships between work passion and work–family interface. Following Aiken and West’s [88] recommendations, variables were mean-centered to minimize multicollinearity before being entered in the analyses. The control variables were entered in Step 1, and an independent variable and a moderating variable were entered in Step 2. In Step 3, the interaction term was entered.

**Fig 3:**
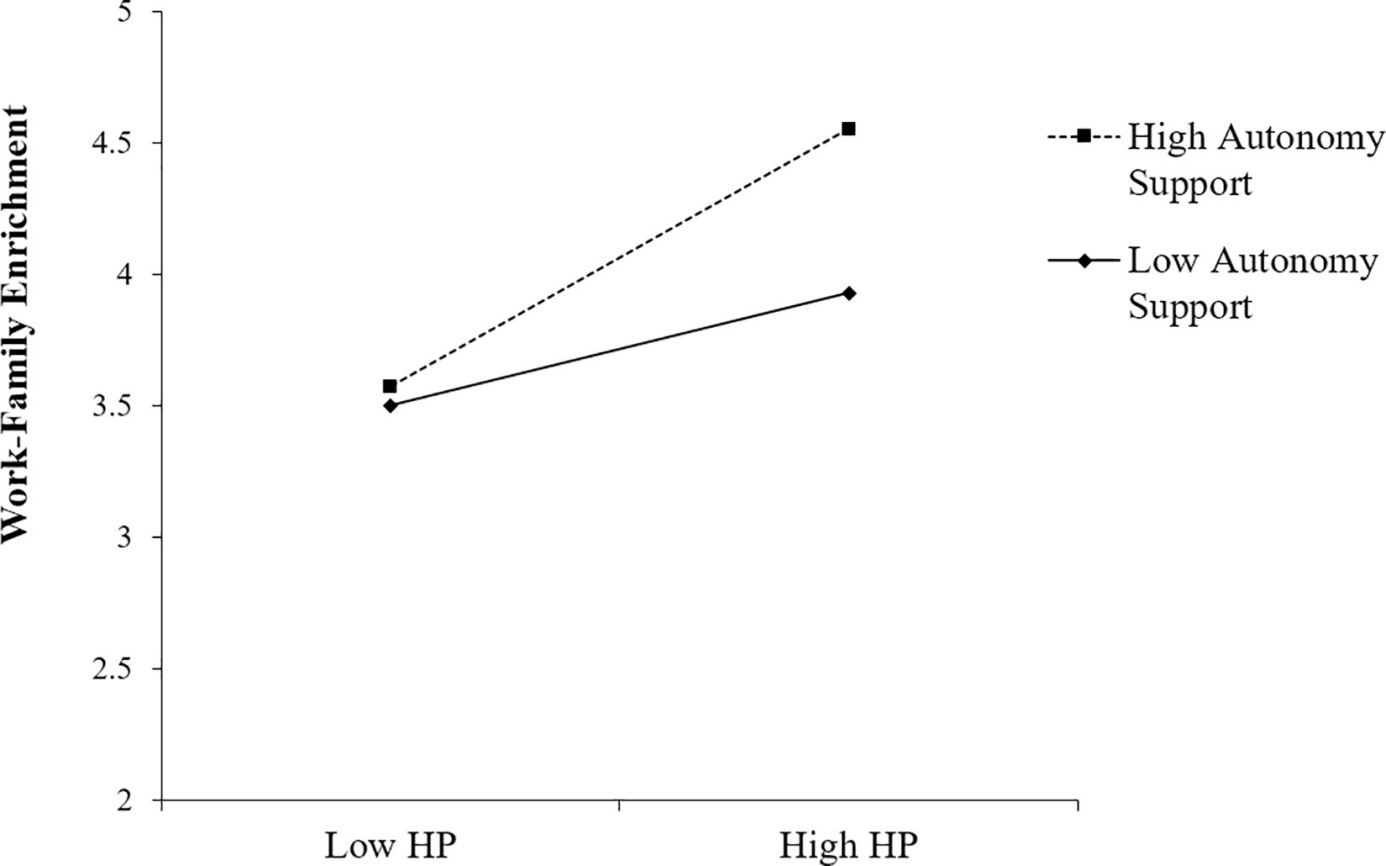
Interaction effect of harmonious work passion and autonomy support on work–family enrichment.

As shown in Model 1 (Table 2), the interaction effect of harmonious work passion at T1 and autonomy support at T1 on work–family enrichment at T2 was significant (*β* = 0.15, *p* < 0.01). To better understand the interaction effect, we plotted the simple slope at one standard deviation above and below the mean of the autonomy support (Fig 3). The slope of the relationship between harmonious work passion and work–family enrichment was relatively strong for employees with high levels of perceived autonomy support (*t* = 5.78, *p* < 0.001), whereas the slope was relatively weak for employees with low levels of perceived autonomy support (*t* = 2.81, *p* < 0.01). Thus, Hypothesis 4a was supported.

**Table 2:** Moderating effects of autonomy support of the relationships between harmonious and obsessive work passion on work–family enrichment and work–family conflict.

Step	Variable	Model 1. WFE		Model 2. WFC	
		*β*	*t*	*β*	*t*
1	Gender	0.02	0.25	−0.18	−2.93**
	Age	0.24	3.86***	−0.02	−0.24
	HP			−0.16	−2.49*
	OP	0.26	4.29***		
*R* ^2^		0.16		0.07	
*F*		15.12***		5.88**	
Δ*R*^2^		0.16		0.07	
2	HP	0.33	5.11***		
	OP			0.45	7.00***
	Autonomy support	0.19	3.13**	−0.05	−0.76
*R* ^2^		0.31		0.22	
*F*		27.63***		24.52***	
Δ*R*^2^		0.16		0.16	
3	HP × Autonomy support	0.15	2.85**		
	OP × Autonomy support			0.02	0.34
*R* ^2^		0.33		0.22	
*F*		8.09**		0.12	
Δ*R*^2^		0.02		0.00	

*Note*. WFE = work–family enrichment; WFC = work–family conflict; HP = harmonious passion; OP = obsessive passion.

As shown in Model 2 (Table 2), the interaction effect of obsessive passion at T1 and autonomy support at T1 on work–family conflict at T2 was not significant (*β* = 0.02, *p* > 0.05). Therefore, Hypothesis 4b was not supported.

## Discussion

The main purpose of this study is to examine the psychological mechanism explaining why and how two distinct types of work passion are differentially associated with career commitment. The results showed that harmonious work passion was positively related to career commitment; however, obsessive work passion was not significantly related to career commitment. Moreover, harmonious work passion and obsessive work passion affect career commitment through different paths regarding the work–family interface. Specifically, the link between harmonious work passion and career commitment was partially mediated by work–family enrichment as a motivational path. The link between obsessive work passion and career commitment was fully mediated by work–family conflict as an impairment path. Finally, we found the moderating effect of the organizational contextual factor, autonomy support, which accentuates the positive impact of harmonious work passion on work–family enrichment.

### Theoretical implications

This study has several theoretical implications. First, this study contributes to the work passion literature by clarifying the differential effects of the types of work passion on career commitment in a non-Western culture. Previously, there was limited research on the impact of the two types of work passion on career commitment that were based on the dualistic model of passion [1,39,92]. We believe this is the first empirical study to test the link between work passion and career commitment using a two-wave data. The results indicate that although harmonious work passion was positively related to career commitment, obsessive work passion was not significantly related to career commitment. These results are consistent with previous research suggesting a positive relationship between harmonious work passion and career consequences when compared with obsessive work passion. For instance, several meta-analyses on work passion revealed that harmonious work passion was positively associated with concentration, flow, intrinsic motivation, and career control, whereas obsessive work passion was not [10,11]. Our findings also extend the work passion literature by enriching our understanding of the differential effects of the two types of work passion on career commitment.

Second, this study suggests the underlying mechanisms explaining why two types of work passion are differentially related to career attitude. Despite numerous studies focusing on the impacts of work passion on work outcomes [26,92], few studies have explored the mediators—particularly the work–family interface—of such relationships [21]. To address this research gap, we examined the mediating roles of work–family enrichment and work–family conflict on the relationship between work passion and career commitment. Our results indicate that harmonious work passion increases the experience of work–family enrichment, subsequently increasing employees’ career commitment. However, obsessive work passion increases the experience of work–family conflict, diminishing employees’ career commitment. This suggests that work–family enrichment partially mediates the relationship between harmonious work passion and career commitment, and that work–family conflict fully mediates the relationship between obsessive work passion and career commitment. These findings empirically demonstrate Chummar et al.’s study [23], which theoretically proposed the mediating effects of work–family interface on work passion—career commitment relationships. Extending prior findings, the present study provides a more comprehensive picture of the distinct pathways through which the two types of work passion impact career attitude.

Finally, this study is the first to investigate interactions between work passion and autonomy support in predicting the perception of the work–family interface. Our findings suggest that autonomy support contributes to fulfilling individuals’ basic needs satisfaction, which leads work passion to more positive consequences based on self-determination theory. Specifically, the positive association between harmonious work passion and work–family enrichment becomes stronger when the level of an organization’s autonomy support is high rather than low. This result supports Gao and Jiang’s findings [27], which suggested that job autonomy accentuates the harmonious work passion–positive outcomes relation. However, contrary to the prediction, the moderating effect of autonomy support on the association between obsessive work passion and work–family conflict was not significant. A possible explanation for this is that the effect of obsessive work passion on negative work–family interaction may not vary depending on the level of autonomy support. Rather than autonomy support, social support can buffer the negative effect of obsessive work passion on work–family conflict. Individuals with highly obsessive work passion are mainly motivated by extrinsic values and gains [93,94]. Therefore, they appear more reactive to situational contingency, such as supervisor or coworker support [13]. Support from supervisors or coworkers can facilitate detachment from their work, which may prevent them from experiencing emotional exhaustion and work–family conflict [23,95]. Previous research also indicated that workplace social support had a relatively greater impact on the inhibition of work–family conflict than autonomy support did [96,97]. Hence, those with obsessive work passion are likely to experience lower work–family conflict when the level of social support is high. Future research should examine the moderating effect of workplace social support on the relation between obsessive work passion and work–family conflict.

### Practical contributions

This study has practical implications as well. First, according to our findings, organizations should be aware of the benefits and prices of distinct types of work passion—harmonious work passion enhances career commitment through work–family enrichment, while obsessive work passion reduces career commitment through work–family conflict. To promote employees’ career commitment, organizations should develop employees’ harmonious passion and hinder obsessive passion. We suggest that organizations train managers to foster their employees’ harmonious passion by identifying their own signature strengths and maximizing the use of their signature strengths [98]. These training programs can facilitate the development of employees’ harmonious passion, which can contribute to their increased positive work–life experience and intrinsic motivation to engage in career commitment.

Second, this study suggests the specific organizational environment that improves the positive effects of work passion in an organizational field. As shown in our study, for workers with harmonious work passion, a higher autonomy support can increase their work–family enrichment, and such joint effect may subsequently accentuate the relationship between harmonious work passion and work–family enrichment. Given that autonomy support can facilitate a supportive work environment that strengthens the positive effects of harmonious work passion [27], organizations need to create an autonomy-supportive work climate. For instance, organizations may redesign the job to ensure employees’ autonomy, encourage employees’ proactivity and feedback seeking behavior, and provide training programs for managers to engage in empowering leadership [99]. Organizations should offer more autonomy support to enable workers with harmonious work passion to experience enhanced work–family enrichment.

### Limitations and future research directions

Despite the aforementioned contributions, the present study also has several limitations. First, we used self-report questionnaires to measure the study variables. Although we collected data at two time points to mitigate the potential impact of common method variance [79], the use of data from a single source may introduce the possibility of common method bias. Future research may use multisource data to reduce the problems associated with common method bias [79]. For example, assessing career commitment that is based on measures from other sources, such as managers or colleagues, would be a better strategy.

Moreover, a time-separated design does not demonstrate a causal relationship between the variables. Longitudinal designs are needed to strengthen causal inferences regarding the influence of work passion on career commitment through work–family interface. A three-wave autoregressive cross-lagged design, for example, could provide strong evidence of causality between the variables.

Third, the data collected from employees in South Korea could present limitations in the generalizability of the present findings. The effects of work passion on work and career outcomes may vary across cultures [1,92,100]. Given that the relationship between work passion and work-related consequences is stronger in individualistic cultures than in collectivistic cultures [10,100], our findings that work–family enrichment and conflict mediate this relationship in a collectivistic South Korean culture are more pronounced. Further research should replicate our study model with a sample of individuals from other collectivistic countries as well. In addition, a cross-cultural study of work passion is needed to identify the generalization of the findings to a diverse set of contexts across individualistic and collectivistic cultures in future studies.

Finally, there may be additional organizational contexts affecting the impact of work passion on work–family enrichment and work–family conflict. Previous research suggests that supportive work–life organizational environments can act as an important moderator that positively influences the work–family interface of passionate workers [23]. Supportive work–life organizations allow employees to psychologically detach from their work and foster their work–life balance by offering adequate resources, such as flexible work schedules, childcare services, and telecommuting or telework [23,101]. Future research should explore the moderating effects of a supportive work–family culture that strengthens the link between harmonious work passion and work–family enrichment and weakens the link between obsessive work passion and work–family conflict.

### Conclusion

This study explored the psychological processes that explain how and why two types of work passion relate differently to career commitment. High levels of harmonious work passion may lead to high levels of work–family enrichment, thereby enhancing career commitment. In contrast, high levels of obsessive work passion may lead to high levels of work–family conflict, thus reducing career commitment. Such results provide support for the differential effects of the two distinct types of work passion on career commitment. Additionally, we found that autonomy support can accentuate the positive impact of harmonious work passion on work–family enrichment. Therefore, interventions designed to promote harmonious work passion and develop an autonomy-supportive work climate would help employees enhance their career commitment.

## Supporting information

S1 Table The means, standard deviations, and correlations between variables. (DOCX)

S1 File Questionnaire. (DOCX)

S2 File Dataset. (XLSX)
