# Supplementary material for: Does work passion benefit or hinder employee’s career commitment? The mediating role of work–family interface and the moderating role of autonomy support
Source: PLoS One. 2022 Jun 3;17(6):e0269298. doi: 10.1371/journal.pone.0269298 (PMC9165830; doi:10.1371/journal.pone.0269298)
Supplement: S1 Table — (DOCX) [file pone.0269298.s001.docx]

S1 Table. The means, standard deviations, and correlations between variables.

|  | Variable | *M* | *SD* | 1 | 2 | 3 | 4 | 5 | 6 | 7 | 8 |
| --- | --- | --- | --- | --- | --- | --- | --- | --- | --- | --- | --- |
| 1. | Gender | 1.51 | 0.50 | $-$ |  |  |  |  |  |  |  |
| 2. | Age | 42.11 | 10.70 | −0.10 | $-$ |  |  |  |  |  |  |
| 3. | HP (T1) | 4.60 | 1.11 | 0.01 | 0.31*** | $-$ |  |  |  |  |  |
| 4. | OP (T1) | 3.59 | 1.14 | −0.03 | 0.27*** | 0.41*** | $-$ |  |  |  |  |
| 5. | WFE (T2) | 4.45 | 1.00 | −0.02 | 0.30*** | 0.50*** | 0.32*** | $-$ |  |  |  |
| 6. | WFC (T2) | 3.59 | 1.45 | −0.20** | −0.05 | −0.19*** | 0.29*** | −0.10 | $-$ |  |  |
| 7. | Career commitment (T2) | 3.02 | 0.77 | 0.03 | 0.29*** | 0.48*** | 0.26*** | 0.40*** | −0.24*** | $-$ |  |
| 8. | Autonomy support (T1) | 4.08 | 1.25 | 0.03 | 0.04 | 0.42*** | 0.28*** | 0.36*** | −0.06 | 0.37*** | $-$ |
| *Note*: HP = harmonious work passion, OP = obsessive work passion, WFE = work-family enrichment, WFC = work-family conflict. **p* < 0.05, ***p* < 0.01, *** *p* < 0.001 | | | | | | | | | | | |
