# Supplementary material for: Does work passion benefit or hinder employee’s career commitment? The mediating role of work–family interface and the moderating role of autonomy support
Source: PLoS One. 2022 Jun 3;17(6):e0269298. doi: 10.1371/journal.pone.0269298 (PMC9165830; doi:10.1371/journal.pone.0269298)
Supplement: S1 File — (DOCX) [file pone.0269298.s002.docx]

S1 File. Questionnaire.

**Work passion**

1. My work is in harmony with the other activities in my life.
2. The new things that I discover about my work allow me to appreciate it even more.
3. My work reflects the qualities I like about myself.
4. My work allows me to live a variety of experiences.
5. My work is well integrated in my life.
6. My work is in harmony with other things that are part of me.
7. I have difficulties controlling my urge to do my work.
8. I have almost an obsessive feeling for my work.
9. My work is the only thing that really turns me on.
10. If I could, I would only do my work.
11. My work is so exciting that I sometimes lose control over it.
12. I have the impression that my work controls me.

**Work-family enrichment**

1. The things you do at work help you deal with personal and practical issues at home.
2. The things you do at work make you a more interesting person at home.
3. Having a good day on your job makes you a better companion when you get home.
4. The skills you use on your job are useful for things you have to do at home.

|  |
| --- |

**Work-family conflict**

1. The demands of my work interfere with my home and family life.
2. The amount of time my job takes up makes it difficult to fulfill family responsibilities.
3. Things I want to do at home do not get done because of the demands my job puts on me.
4. My job produces strain that makes it difficult to fulfill family duties.
5. Due to work-related duties, I have to make changes to my plans for family activities.

**Career commitment**

1. If I could go into a different profession other than this profession which paid the same, I would probably take it.
2. I definitely want a career for myself in this profession.
3. If I could do it all over again, I would not choose to work in this profession.
4. If I had all the money I needed without working, I would probably still continue to work in this profession.
5. I like this vocation too well to give it up.
6. This is the ideal vocation for a life work.
7. I am disappointed that I ever entered this profession.

**Autonomy support**

1. I feel that my manager provides me choices and options.
2. I feel understood by my manager.
3. My manager conveys confidence in my ability to do well at my job.
4. My manager encourages me to ask questions.
5. My manager listens to how I would like to do things.
6. My manager tries to understand how I see things before suggesting a new way to do things.
